# Case report: Malignant chemodectoma with hepatic metastasis in a cat

**DOI:** 10.3389/fvets.2023.1216439

**Published:** 2023-07-18

**Authors:** Shakirat Adeola Adetunji, Kaiwen Chen, Justin Thomason, Franco Matias Ferreyra

**Affiliations:** ^1^Kansas State Veterinary Diagnostic Laboratory, Diagnostic Medicine and Pathobiology, Kansas State University, Manhattan, KS, United States; ^2^Department of Clinical Sciences, College of Veterinary Medicine, Kansas State University, Manhattan, KS, United States

**Keywords:** aortic body tumor, chemodectoma, feline, malignant, metastasis

## Abstract

A 10-year-old, male-neutered, domestic short-hair cat was examined at the Veterinary Health Center Emergency Service at Kansas State University for a one-day history of dyspnea. Prior to thoracocentesis, sedation was provided. The cat stopped breathing after sedation and went into cardiac arrest. Cardiopulmonary resuscitation (CPR) was unsuccessful. At necropsy, there was severe pleural effusion and bilateral pulmonary atelectasis. The myocardium of the atria and ventricles, and tunica adventitia of coronary vessels, pulmonary artery, and aorta, had pale, firm, multinodular masses ranging from 0.3 to 0.5 cm in diameter. Multiple nodules were also present in the liver. Multifocally expanding the epicardial fat and compressing the underlying epicardium, infiltrating, and expanding the myocardium, and expanding the walls of major vessels, there was a multinodular, unencapsulated, densely cellular neoplasm composed of polygonal epithelial cells arranged in nests and packets and supported by a fine fibrovascular stroma. The nodules in the liver had similar histologic features. In this case, neoplastic cells at the primary and metastatic sites were intensely immunoreactive to synaptophysin, variably reactive to chromogranin A, and negative for neuron specific enolase, cytokeratin, vimentin, thyroglobulin, and smooth muscle actin. The gross, histologic, and immunohistochemical findings support the diagnosis of chemodectoma, with metastases to the liver. Synaptophysin and chromogranin A were the most useful immunohistochemical markers to diagnose malignant chemodectoma in this cat.

## Introduction

Primary cardiac neoplasms are rare in domestic cats and very few reports exist in the literature. Most commonly reported cardiac neoplasms in these animals include lymphoma, hemangiosarcoma, rhabdomyosarcoma, ganglioneuroma, metastatic tumors, and less frequently, aortic and carotid body tumors (paraganglioma/chemodectoma) (1–14). Unlike cats, chemodectomas are common in dogs, especially in the brachycephalic breeds, and there is an abundant literature detailing the clinical and pathologic features, as well as management of this neoplasm (2, 15–17).

Chemodectoma is a generally benign neoplasm composed of neoplastic chemoreceptor cells and are often benign. Chemoreceptor cells are present in several tissues of the body, including aortic and carotid bodies located in the submandibular and cranial cervical regions and adventitia of major vessels at the base of the heart, pancreas, eyes, ear, and some cranial nerves (18). Chemoreceptor cells detect changes in pH, carbon dioxide, and oxygen tension; thus, they aid in the regulation of physiologic parameters including respiration and blood circulation (19, 20). These cells are similar to adrenal medullary cells; however, they are negative for chromaffin stain; hence, neoplasms of chemoreceptor cells are designated chemodectoma or nonchromaffin paraganglioma (17). In domestic animals, neoplasms of chemoreceptor tissue commonly develop in the aortic and carotid bodies, but aortic body neoplasms occur more frequently (2, 12). The pathogenesis of chemodectoma is not yet established; however, several factors have been implicated including genetic predisposition in some breeds of dogs (brachycephalic), and especially chronic hypoxia, given that chemoreceptor cells regulate respiration and blood circulation parameters (21). Additionally, studies have shown an increased incidence of chemodectomas in humans and animals (mostly brachycephalic dogs and cattle) that live in high altitude environments that commonly induce chronic hypoxia (17, 22, 23).

Chemodectomas are known to be functionally inactive, and most of the observed clinical signs are associated with the space-occupying effects of the tumor or other complications such as tachypnea/dyspnea secondary to pleural effusion or dysphagia as a result of esophageal compression (24). There has been one case of tachypnea/dyspnea due to pleural effusion secondary to chemodectoma in a cat. As such, clinical diagnosis of slow-growing heart base neoplasms is difficult until they reach a critical size or start to cause secondary pathological changes. Therefore, even though chemodectoma cells are fragile and exfoliate well, the diagnosis is often made during necropsy.

In dogs, there is minimal risk of metastasis but this is common (1, 16, 25, 26). Surgical removal, chemotherapy and/or radiation therapy are the typical treatment options in humans and dogs (22). Reported treatment options for cats include thoracocentesis, chemotherapy, and surgical cytoreduction. Survival time for heart base chemodectomas ranges from euthanasia immediately after diagnosis to 19 months (27).

To date, very few cases of chemodectoma in cats are reported in literature, and much less documentation of local or distance metastasis of the neoplasm is available (2, 22, 24, 27). With paucity of literature regarding the incidence and predisposing factors of this neoplasm in cats, advances in early diagnosis and management of chemodectoma in cats remain to be further characterized and investigated. This report documents a case of metastatic chemodectoma with emphasis on common immunological diagnostic markers useful for the diagnosis of this entity in a domestic adult cat.

## Methods and results

A 10-year-old, male-neutered, domestic short-hair cat was referred to the Kansas State University Veterinary Health Center Emergency Service for evaluation of a one-day history of dyspnea with previous reported episodes of wheezing during the fall season. The cat was otherwise healthy and respiratory clinical signs were previously attributed to exposure to wildfire smoke. On arrival, the cat had an open mouth breathing and was placed in an oxygen cage for supportive care. After stabilization, a more thorough examination revealed reduced bilateral lung sounds, but no murmur was evident on auscultation. Thoracic focused assessment with sonography for trauma, triage, and tracking testing (TFAST) revealed moderate bilateral pleural effusion more markedly affecting the right hemithorax. The cat was given butorphanol (0.23 mg/kg) intramuscularly and furosemide (3 mg/kg) intravenously. The respiratory rate decreased after the administration of medications, but the patient was still orthopneic. Subsequently, Midazolam (0.2 mg/kg) and Alfaxalone (0.75 mg/kg) were administrated intravenously to facilitate therapeutic thoracocentesis. Although sedated, the respiratory rate of the patient continued to decrease, and he developed cardiac and pulmonary arrest shortly after. Cardiopulmonary resuscitation (CPR) was performed, and spontaneous breathing returned. The cat developed cardiac arrest again after about 10 min and additional CPR was performed but was not successful.

**Figure 1:**
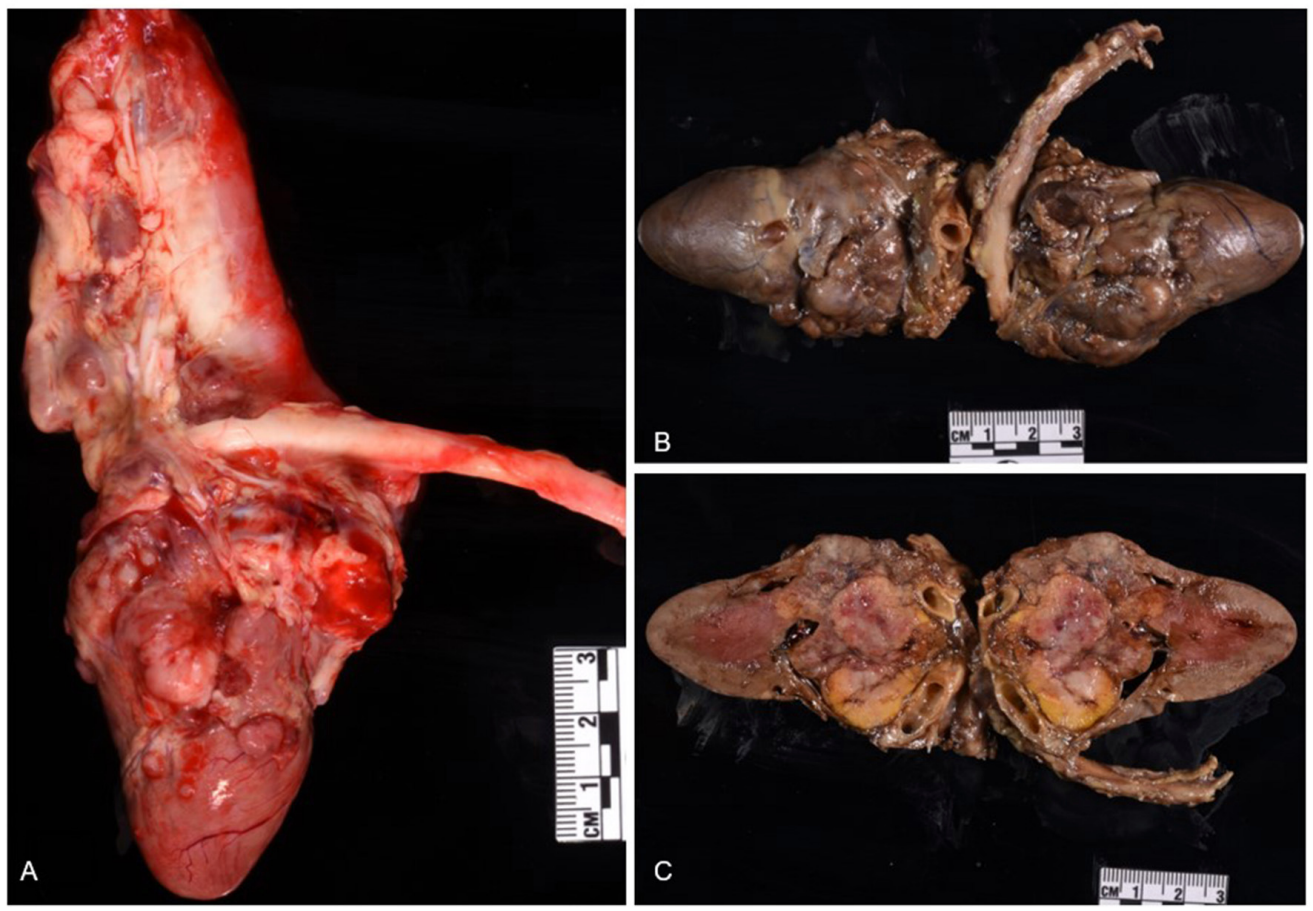
Chemodectoma in the heart of a domestic short-hair cat. The neoplasm effaced the right and left atria, atrioventricular valves, and major blood vessels of the heart. Fresh heart **(A)**; formalin-fixed heart **(B, C)**.

At necropsy, the animal was in good nutritional condition with adequate subcutaneous and intra-abdominal fat, as well as adequate skeletal muscling. The pleural cavity contained approximately 700 mL of thick, clear, fluid. Bilaterally, the lungs were diffusely atelectatic. The pericardium was firmly adherent to the right and left atria and the pericardial cavity contained approximately 5 mL of free, dark red, slightly viscous fluid. The epicardium, epicardial adipose tissues, interatrial septum, the tunica adventitia of coronary vessels, pulmonary artery, and aorta, had multiple, adherent, firm, pale, multinodular masses ranging from 0.3 to 0.5 cm in diameter, which were red-to-white and marbled on cut surfaces (Figure 1A). After formalin-fixation, a longitudinal section of the heart revealed multiple, firm, yellow-rimmed, marbled masses with red-white centers that effaced the aortic, pulmonic, and atrioventricular valves, and both atria (Figures 1B, C). Affecting all liver lobes were multiple, tan, firm, variably sized nodular masses (the largest nodule was 1.5 × 1 × 0.4 cm) that extended into the parenchyma on cut sections. Other macroscopic findings included diffusely small shrunken (3.5 cm in length compared to the right, 5 cm), and irregular left kidney with a 1 cm-wide infarct extending from the cortex to the medulla.

**Figure 2:**
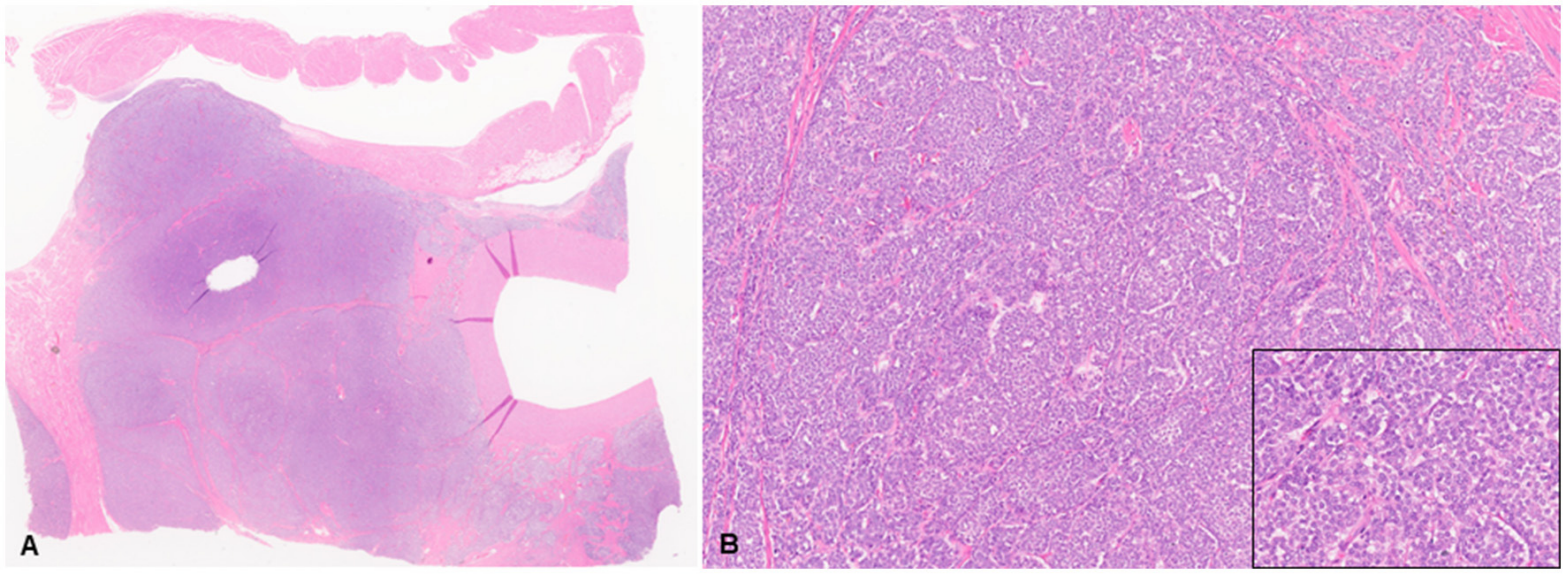
Chemodectoma in a domestic cat, pulmonary artery and right atrium. Neoplastic cells expand and efface the tunica adventitia of the pulmonary artery, adjacent connective and adipose tissues, and invade the right atrium. Hematoxylin and Eosin stains (H&E). Sub-gross view **(A)** and higher magnification **(B)**. Inset: 400X H&E.

**Figure 3:**
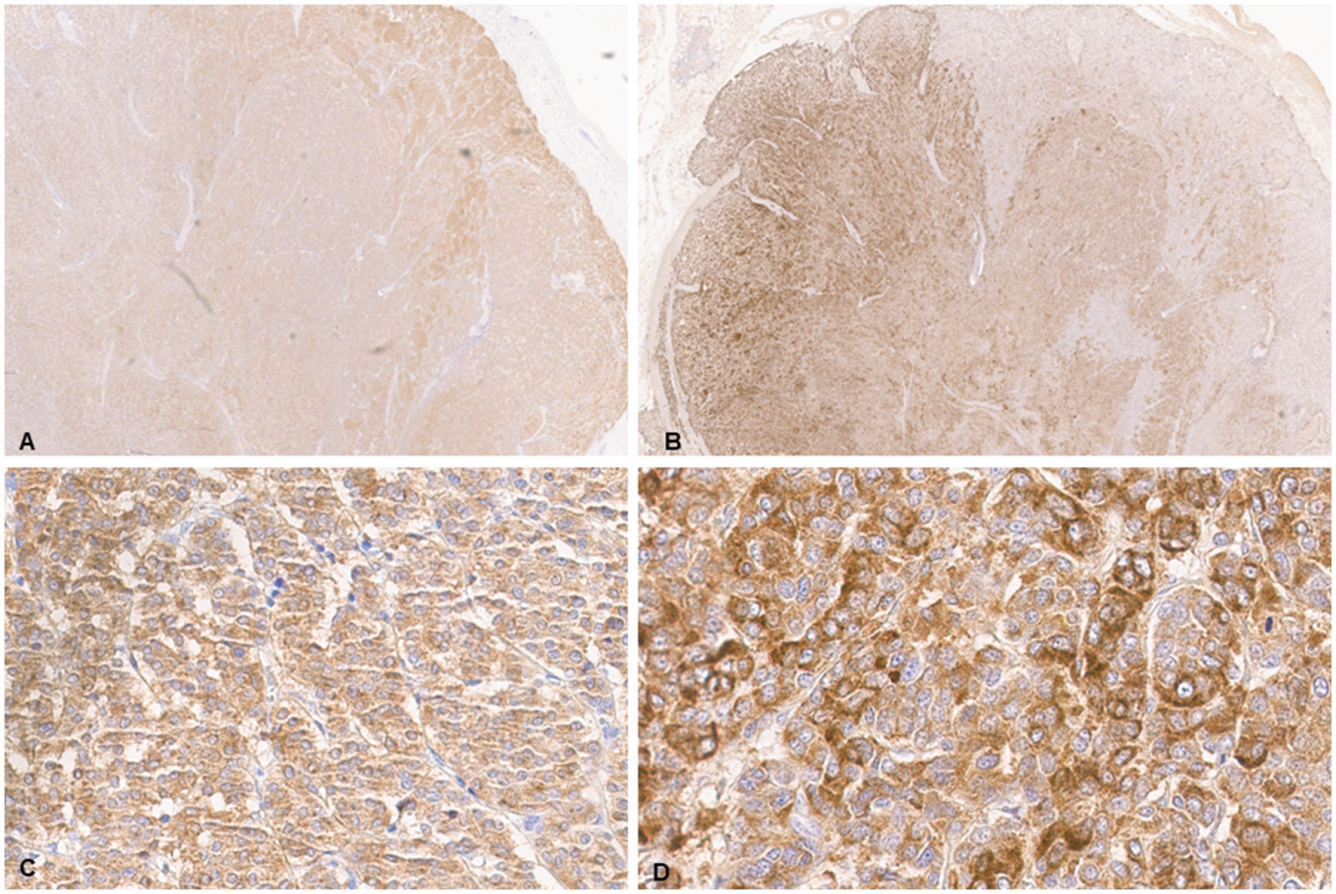
Chemodectoma in a domestic cat, heart. Immunohistochemical analysis (IHC). Neoplastic cells stained strongly for synaptophysin **(A, C)** and chromogranin A **(B, D)**.

Histologic evaluation was performed on routinely processed formalin-fixed, paraffin-embedded tissue sections (5 μm) stained with hematoxylin and eosin (H&E). All H&E reagents were purchased from StatLab, McKinney, Texas, US. Microscopic evaluation of the masses in the heart revealed an infiltrative, non-encapsulated, poorly circumscribed, densely cellular multilobulated neoplasm composed by polygonal cells arranged in nests and packets and supported by a fine fibrovascular stroma (Figure 2). Neoplastic cells had variably distinct cell borders and moderate amounts of finely granular eosinophilic cytoplasm, single round to oval nuclei with finely stippled chromatin and one variably distinct nucleolus. There was mild anisocytosis and moderate anisokaryosis with rare karyomegaly. There were 22 mitotic figures in 2.37 mm^2^ (10 consecutive 400X fields). The neoplasm had multifocal areas of hemorrhage and necrosis characterized by cytoplasmic hypereosinophilia and nuclear pyknosis and karyorrhexis; and was rarely infiltrated by moderate numbers of lymphocytes and macrophages. Epicardial fat, tunica adventitia, media and intima of large arteries were also infiltrated and effaced by nests and packets of neoplastic cells. In the liver, nodules were consistent with metastatic dissemination from the heart neoplasm based on similar histologic and morphological features. Immunohistochemical analysis of sections of the heart and liver neoplasm was performed at Kansas State Veterinary Diagnostic Laboratory following internal validated protocols. All immunostains are listed in Table 1 and were performed using a Leica Bond Rxm IHC Stainer (Leica Biosystems, Deer Park, Illinois, US). Briefly, antigen retrieval was performed using citrate (pH 6.0; 10–20 min) and EDTA buffers (pH 9.0; 10–20 min). Subsequently, sections were incubated for 15 min with primary antibodies against cytokeratin (mouse anti-AE1/AE3; 1:100; Leica Biosystems, Illinois, US), synaptophysin (mouse anti-sy38; 1:100; Dako/Agilent, Santa Clara, CA, US), chromogranin A/SP-1 (rabbit polyclonal; 1:500; Refine detection kit, ImmunoStar, Hudson, Wisconsin, US), neuron specific enolase (mouse, clone L1830, Refine Red Detection kit by Leica–Deer Park, IL), thyroglobulin (rabbit anti-EPR9730; 1:7,000; Abcam, Boston, US), vimentin (mouse anti-SRL33; prediluted; Leica Biosystems, Deer Park, Illinois, US), and smooth muscle actin–SMA (mouse anti-alpha sm-1; prediluted; Leica Biosystems, Deer Park, Illinois, US). Tissue sections were subsequently incubated for 25 min with secondary anti-rabbit Poly-HRP IgG, or anti-mouse PowerVision Poly-AP Mouse IgG from Refine Detection staining kits provided by the manufacturer (Leica Biosystems, Deer Park, Illinois, US). Finally, sections were counterstained with Gill's hematoxylin (StatLab, McKinney, Texas, US). Sections of normal tissues from other animals were used for positive control; skin for cytokeratin, thyroid for thyroglobulin, adrenal gland for chromogranin and synaptophysin, urinary bladder for SMA, and a dermal malignant fibrous histiocytoma was used for vimentin. Approximately 100% and 70% of neoplastic cells were strongly positive for synaptophysin and chromogranin A, respectively (Figure 3). Neoplastic cells were negative for cytokeratin AE1/AE3, vimentin, thyroglobulin, and smooth muscle actin. Similar observations were noted in the metastatic neoplastic cells in the hepatic parenchyma.

**Table 1:** Primary and secondary antibodies used for immunohistochemical analysis of neoplastic cells in the tissues of a domestic cat with malignant chemodectoma.

**Antibody**	**Species**	**Clone**	**Dilution**	**Retrieval method**	**Polymer**	**Staining kit**	**Antibody manufacturer**
Chromogranin A/SP-1	Rabbit	Polyclonal	1:500	EDTA pH 9.0 10 min, 100°C	Anti-rabbit Poly-HRP-IgG (from kit)	Refine detection	ImmunoStar (Hudson, Wisconsin)
Cytokeratin	Mouse	AE1/AE3	1:100	EDTA pH 9.0 20 min, 100°C	PowerVision Poly-AP Mouse IgG	Refine red detection	Leica (Deer Park, IL)
Smooth muscle actin	Mouse	alpha sm-1	Predilute	Citrate pH 6.0 10 min, 100°C	PowerVision Poly-AP Mouse IgG	Refine red detection	Leica (Deer Park, IL)
Synaptophysin	Mouse	SY38	1:100	Citrate pH 6.0 20 min, 100°C	PowerVision Poly-HRP anti-Mouse IgG	Refine detection	Dako/Agilent (Santa Clara, CA)
Thyroglobulin	Rabbit	EPR9730	1:7,000	EDTA pH 9.0 10 min, 100°C	Anti-rabbit Poly-HRP-IgG (from kit)	Refine detection	abcam (Boston, MA)
Vimentin	Mouse	SRL33	Predilute	EDTA pH 9.0 20 min, 100°C	PowerVision Poly-AP Mouse IgG	Refine red detection	Leica (Deer Park, IL)
Neuron specific enolase	Mouse	L1830	Predilute	EDTA pH 9.0 20 min, 100°C	PowerVision Poly-AP Mouse IgG	Refine red detection	Leica (Deer Park, IL)

## Discussion

This report describes primary cardiac and vascular chemodectoma with hepatic metastatic dissemination in a domestic cat. As mentioned previously, primary heart neoplasms are rarely reported in domestic cats. However, chemodectoma, hemangiosarcoma, ectopic thyroid, and parathyroid carcinoma have been documented (2, 8). Therefore, characterization of cardiac neoplasms using gross morphology, histology, and immunohistochemical analysis are crucial to differentiating neoplastic diseases affecting the heart. In this case, the primary gross differential diagnosis of the neoplasm was determined to be a chemodectoma based on the location of the tumor at the heart base and the major vessels of the heart. Further testing included microscopic evaluation and positive immunoreactivity to synaptophysin, and chromogranin A, all of which supported the diagnosis of chemodectoma. Neoplastic cells had negative immunoreactivity for thyroid transcription factor 1 and thyroglobulin (thyroid and pulmonary tumors), smooth muscle actin (smooth muscle tumors), vimentin (mesenchymal tumors), cytokeratin AE1/AE3 (epithelial tumors).

Chemodectomas are non-chromaffin neuroendocrine tumors arising from neoplastic transformation of chemoreceptor cells located within the tunica adventitia of major blood vessels, frequently at the bifurcation of the common carotid arteries (carotid body) and along the aortic arch (aortic body) (28, 29). Chemoreceptor cells are specialized sensory receptor cells with known regulatory functions in the cardiorespiratory system through the monitoring of pH, carbon dioxide, and oxygen concentrations (19, 20).

In general, chemodectomas are non-functional, space-occupying, and locally invasive masses, with a low reported metastatic potential (up to 22% in canine tumors) (2, 28). Similarly, in feline, five out of the 13 case reports have reported metastasis to other tissues within the thoracic cavity including lungs and diaphragm (20, 27, 30, 31). Carotid body neoplasms are known to be more malignant and often metastasize to other organs than aortic body neoplasms. Chemodectomas are usually present as a single mass at the base of the heart, most often occurring between the aorta and pulmonary artery, the aorta and right atrium, or between the pulmonary artery and left atrium (2, 24), which was the location in this case. The most common sites of metastatic chemodectoma include the regional lymph nodes, lungs, liver, adrenal glands, and brain (23, 24, 28). Metastasis in this cat was to the liver, affecting ~20% of the total liver volume. Nothing is known about the tropism to different tissues. However, the liver is a common site for metastasis of several neoplasms. The factors that make one organ more susceptible to metastasis of chemodectoma are currently unknown.

The cat in this report was 10 years old which falls within the common age range at which chemodectomas have been reported (10–15 years). In general, older animals are more susceptible to neoplastic diseases because of the inherent chronic exposure to environmental insults, inevitable accumulation of DNA damage, or impaired DNA repair mechanisms. In canines, metastasis to the liver, lung, kidney spleen, bone marrow, lymph nodes (15–17) has been previously reported. One study indicated that a higher neoplasm weight-to-body ratio correlates with malignant potential of aortic body neoplasms in dogs; however, the hallmarks of malignancy including mitotic index and nuclear pleomorphism, as well as immunohistochemical labeling intensity of neoplastic cells are not sensitive determinants of malignant aortic body tumors (26).

Expression of neuron specific enolase (NSE) was present in all neoplastic cells in canine aortic body neoplasm irrespective of metastasis (26). However, in the current case of feline chemodectoma, the neoplastic cells were largely negative for NSE marker, indicating the non-sensitivity of chemodectoma in cats for NSE. Interestingly, a prior feline chemodectoma case report also documented negative staining for NSE markers (24) while another reported variable staining intensity indicating that NSE is only partially reliable as a marker for the diagnosis of chemodectoma in domestic cats (27). Another important finding in the current case report is that neoplastic cells were intensely immunoreactive to synaptophysin, a consistent finding in other reports (24, 27), demonstrating that synaptophysin is an important diagnostic marker for chemodectoma in domestic cats.

In this case, the exact tissue origin of the neoplasm is unknown (carotid vs. aortic body vs. atrium). The respiratory distress in the cat is attributed to pleural effusion and bilateral pulmonary atelectasis, secondary to the neoplasm. The neuroendocrine origin of the neoplasm was confirmed by positive immunoreactivity to synaptophysin and chromogranin A. Interestingly, neoplastic cells in the liver were also strongly positive for synaptophysin and chromogranin A, and negative for the other previously stated immunohistochemical markers. Thyroid, epithelial, and mesenchymal tumors were ruled out with the specific immunohistochemical markers as previously described (Table 1). Taken together, the gross morphology, histologic features, and immunohistochemical reactivity supported the diagnosis of chemodectoma, with metastasis to the liver. The chylothorax observed in this case was likely secondary to neoplasia-associated thoracic duct rupture (not observed during autopsy) or impedance of lymphatic outflow at the level of lymphatic venous anastomosis (32, 33). Drainage of the chylothorax by an intrathoracic drainage catheter in a dog with heart base neoplasm has been shown to improve survival in a dog (32).

In conclusion, this case report describes the clinical, gross morphologic and histopathologic features, as well as immunoreactivity of neoplastic cells in a metastatic feline chemodectoma. This neoplasia should be considered as a differential diagnosis in cases of sudden onset of respiratory distress, pleural effusion, pulmonary atelectasis, and a space occupying mass in the thoracic cavity in domestic cats. Immunohistochemical analyses indicate that synaptophysin and chromogranin A are more useful ancillary tests in the diagnosis of feline chemodectomas. Based on this current report and the review of relevant literature, a recommended immunohistochemical panel to support the diagnosis of chemodectoma in domestic cats include synaptophysin and chromogranin A (Table 2). The neoplasm in this case was considered non-functional based on the absence of tumor-specific clinical signs. Further studies are required to investigate and predict metastatic potential of chemodectoma in cats and premortem diagnostic markers of the disease.

**Table 2:** Chemodectoma in domestic cats. Summary of select relevant literature in the past 20 years.

**Diagnostic methods**	**Adetunji et al. (unpublished)**	**Martinez et al. (34)**	**Saunders et al. (27)**	**Hansen et al. (24)**	**Paltrinieri et al. (35)**
Clinical evaluation	Dyspnea, pleural effusion, and sudden death	Chylous pleural effusion	Pleural effusion	Pleural effusion, intrathoracic mass by computed tomography, and sudden death	Dyspnea and pleural effusion
Cytology	None	None	None	Fine needle aspiration	None
Histopathologic evaluation	Affected tissues include heart, great vessels, tracheobronchial and mediastinal lymph nodes, and liver	Cranial vena cava and azygous vein	Heart	Aortic body tumor cells in the lungs, pleura, intercostal muscles, and kidneys	Heart
Metastasis	Liver	Not reported	Not reported	Lungs, pleura, intercostal muscles, and kidneys	Not reported
Immunohistochemical panel	Chromogranin A, synaptophysin, neuron specific enolase, thyroglobulin, smooth muscle actin, vimentin, and cytokeratin AE1/AE3	Thyroglobulin only	Chromogranin A, synaptophysin, neuron specific enolase, cytokeratin AE1/AE3, calcitonin, and thyroglobulin	Chromogranin A, synaptophysin, neuron specific enolase, and cytokeratin AE1/AE3	Chromogranin A, synaptophysin, neuron-specific enolase, vimentin, cytokeratin, alpha smooth muscle actin, glial fibrillary acidic protein, thyroglobulin, and calcitonin
Immunoreactivity	Neoplastic cells have intense immunopositivity for synaptophysin and chromogranin A	Neoplastic cells were negative for thyroglobulin	Neoplastic cells were moderately to strongly positive for chromogranin A, synaptophysin, and neuron specific enolase; and were negative for cytokeratin AE1/AE3, calcitonin, and thyroglobulin	Neoplastic cells were moderately positive for synaptophysin and negative for chromogranin A, neuron specific enolase, and cytokeratin AE1/AE3	Neoplastic cells were positive for chromogranin A and synaptophysin; faintly positive for neuron-specific enolase; and were negative for vimentin, cytokeratin, alpha smooth muscle actin, glial fibrillary acidic protein, thyroglobulin, and calcitonin

## Data availability statement

The original contributions presented in the study are included in the article/supplementary material, further inquiries can be directed to the corresponding author.

## Ethics statement

Ethical review and approval was not required for the study of animals in accordance with the local legislation and institutional requirements. Written informed consent from the owners for the participation of their animals was not required in accordance with the national legislation and the institutional requirements.

## Author contributions

SA, KC, and FMF wrote, reviewed, and edited the manuscript. JT reviewed and edited the manuscript. All authors contributed to the article and approved the submitted version.
